# Mineralization and morphology of peri-implant bone around loaded and unloaded dental implants retrieved from the human mandible

**DOI:** 10.1007/s10006-023-01175-1

**Published:** 2023-09-04

**Authors:** Fausto Zamparini, Maria Giovanna Gandolfi, Andrea Spinelli, Mauro Ferri, Giovanna Iezzi, Daniele Botticelli, Carlo Prati

**Affiliations:** 1 Laboratory of Biomaterials and Oral Pathology, Department of Biomedical and Neuromotor Sciences, School of Dentistry, University of Bologna, 40126 Bologna, Italy; 2 Endodontic Clinical Section, Department of Biomedical and Neuromotor Sciences, School of Dentistry, University of Bologna, 40126 Bologna, Italy; 3 School of Dentistry, Corporación Universitária Rafael Núñez, 130001 Cartagena, de Indias, Colombia; 4 Department of Medical Oral and Biotechnological Sciences, University of Chieti/Pescara, 66013 Chieti, Italy; 5 ARDEC Academy, 47923 Rimini, Italy

**Keywords:** Dental implants, ESEM-EDX, Retrieved implants, Peri-implant bone, Loading, Mineralization degree

## Abstract

**Purpose:**

Limited data is reported regarding the bone mineralization around dental implants in the first months from insertion. The study analyzed the peri-implant bone around loaded and unloaded implants retrieved from human mandible after 4 months from placement.

**Method:**

The composition and mineralization of human bone were analyzed through an innovative protocol technique using Environmental-Scanning-Electron-Microscopy connected with Energy-Dispersive-X-Ray-Spectroscopy (ESEM/EDX). Two regions of interest (ROIs, approximately 750×500 μm) for each bone implant sample were analyzed at the cortical (Cortical ROI) and apical (Apical ROI) implant threads. Calcium, phosphorus, and nitrogen (atomic%) were determined using EDX, and the specific ratios (Ca/N, P/N, and Ca/P) were calculated as mineralization indices.

**Results:**

Eighteen implant biopsies from ten patients were analyzed (unloaded implants, *n*=10; loaded implants, *n*=8). For each ROI, four bone areas (defined bones 1–4) were detected. These areas were characterized by different mineralization degree, varied Ca, P and N content, and different ratios, and by specific grayscale intensity detectable by ESEM images. Bony tissue in contact with loaded implants at the cortical ROI showed a higher percentage of low mineralized bone (bone 1) and a lower percentage of remodeling bone (bone 2) when compared to unloaded implants. The percentage of highly mineralized bone (bone 3) was similar in all groups.

**Conclusion:**

Cortical and apical ROIs resulted in a puzzle of different bone “islands” characterized by various rates of mineralization. Only the loaded implants showed a high rate of mineralization in the cortical ROI.

**Supplementary Information:**

The online version contains supplementary material available at 10.1007/s10006-023-01175-1.

## Introduction

Histological and biochemical investigations of peri-implant bone tissue indicate great cell turnover with different cells involved in neo-angiogenesis, vascularization, bone formation, and remodeling, which are responsible for new bone morphology [1–7].

Osseointegrated dental implants should possess thigh and strict bone tissue around the subcrestal threads [4, 8]. For this reason, bone implant contact is usually calculated on histological samples [9–11]. Bone in contact with the implant surface and bone around the implant can be influenced by mechanical loads, particularly in the early months after insertion [4].

In recent years, different animal models have been used to study bone morphology around implants immediately after insertion and during bone healing [12–17]. However, animal models usually have discrepancies when directly translated to humans. In some studies, no occlusal load was applied to the implants [14–17]. In other studies, femoral or tibial bones were used, with obvious biological and biomechanical differences [12, 13, 18, 19].

Several studies have considered retrieved human dental implants as samples to obtain more information on the morphology of the bone around and in contact with the implant surface in clinical conditions [1–3, 5–7, 10, 20, 21]. These morphological studies demonstrated great remodeling activity with many modifications of bone and with an active role of the blood cloth and other tissues [1–3, 5–7]. The roles of necrotic bone debris, smear layer, and fibrous tissue have only partially been described in these studies [3, 5, 20, 21]. Moreover, limited data is reported regarding the bone mineralization and composition around dental implants in the first months from their insertion in human mandible.

Environmental Scanning Electron Microscopy (ESEM) connected with Energy Dispersive X Ray Spectroscopy (EDX) allows to evaluate the element content (atomic %) of inorganic and organic samples. Recently, a protocol to investigate the bone mineralization around retrieved dental implants has been conceived [22, 23]. This analysis has been used to investigate the composition of bone around implants using histological preparations from human biopsies, providing a direct comparison with the optical information from the same histological preparation [22–25].

The aim of this study was to investigate the bone mineralization and morphology around dental implants retrieved from the human mandible after 4 months under two different loading conditions. One implant group was loaded after 2 months, and the other group was left unloaded.

## Methods

Detailed information on the clinical procedures have been reported in a previous paper [5]. The protocol of the study was approved on October 8, 2014, (CURN 07- 2014) by the Ethical Committee of the Corporación Universitária Rafael Núñez, Cartagena de Indias, Colombia.

Sample size calculation has been performed following a previous paper [26]. PS Power and Sample Size Calculations software (Version 3.0) was used [27]. In order to reach Power analysis 0.9 and *α* error 0.05, a minimum of 12 implants per group were necessary. This number was further increased to 16 to compensate for any losses or problems with biopsies analyses.

All procedures were made according to the recommendations of the declaration of Helsinki [28]. The study was designed in accordance with the CONSORT guidelines [29].

Healthy, nonsmoker volunteers were recruited and had to fulfill strict inclusion criteria [5]. Patients received two mini-implants (5-mm height, 3.5-mm width, Sweden & Martina, Due Carrare, Padova, Italy) in the posterior region of the mandible (molar areas). All surgeries were conducted on fully-healed crests in sites with adequate bone volumes and height and without the need for any bone graft procedures.

All implants were characterized by a ZirTi surface (Sweden & Martina, Due Carrare, Padova, Italy), obtained by zirconium microspheres sandblasting and by hydrofluoric acid, sulfuric acid, and hydrochloric acid etching treatments [30].

All surgical procedures were performed by a skilled operator. After local anesthesia, full-thickness flaps were raised, and an osteotomy performed with a series of drills with increasing diameter. Final alveolar socket preparation was 2.8 mm in width in the apical portion, 3.0 mm in width in the cortical portion, and 5.0 mm in depth. Implants were placed with the cortical margin flush to the bony crest. A healing screw was positioned to allow a non-submerged placement.

Implant loading protocol was selected according to Gallucci et al. [31]. After 2 months, each patient had one implant randomly loaded with a cement-retained crown (loaded group); the other implant was left unloaded (unloaded group). In loaded group, cemented resin crowns were inserted and maintained up to 4 months from placement. Crowns were designed only to have vertical contacts, which were checked at 3 months and 4 months from implant placement [5]. In the unloaded group, the healing screw was maintained up to 4 months from placement.

### Histological biopsies preparation

Detailed information on the histological preparation has been reported in a previous paper [5]. After 4 months from installation, mini-implants were removed with a trephine bur. Biopsy specimen containing the implants were fixed in 10% buffered formalin immediately after retrieval.

The specimens were first dehydrated in alcohol and then included in a glycol methacrylate resin (Technovit 7200 VLC; Kulzer, Wehrheim, Germany). Subsequently, they were polymerized and sectioned using a diamond steel disc along the major axis of the implants at approximately 150 mm and ground to about 30 μm. The sections were stained with acid fuchsin and toluidine blue.

### Optical microscopy and ESEM-EDX microanalysis

Optical microscopy (OM) was used to identify the peri-implant bone morphology.

The biopsies were then placed on the ESEM stub and examined without any prior preparation (uncoated samples) following the previously-validated protocol set by Gandolfi et al. [22–25]. Operative parameters were established as follows: low vacuum 100 Pascal, accelerating voltage of 20–25 kV, working distance of 8.5 mm, and 133-eV resolution in Quadrant Back-Scattering Detector mode (0.5 wt% detection level, amplification time 100 μs, measuring time 60 s). All images were taken with the same parameters and at the same magnification on each implant ROI.

**Fig. 1:**
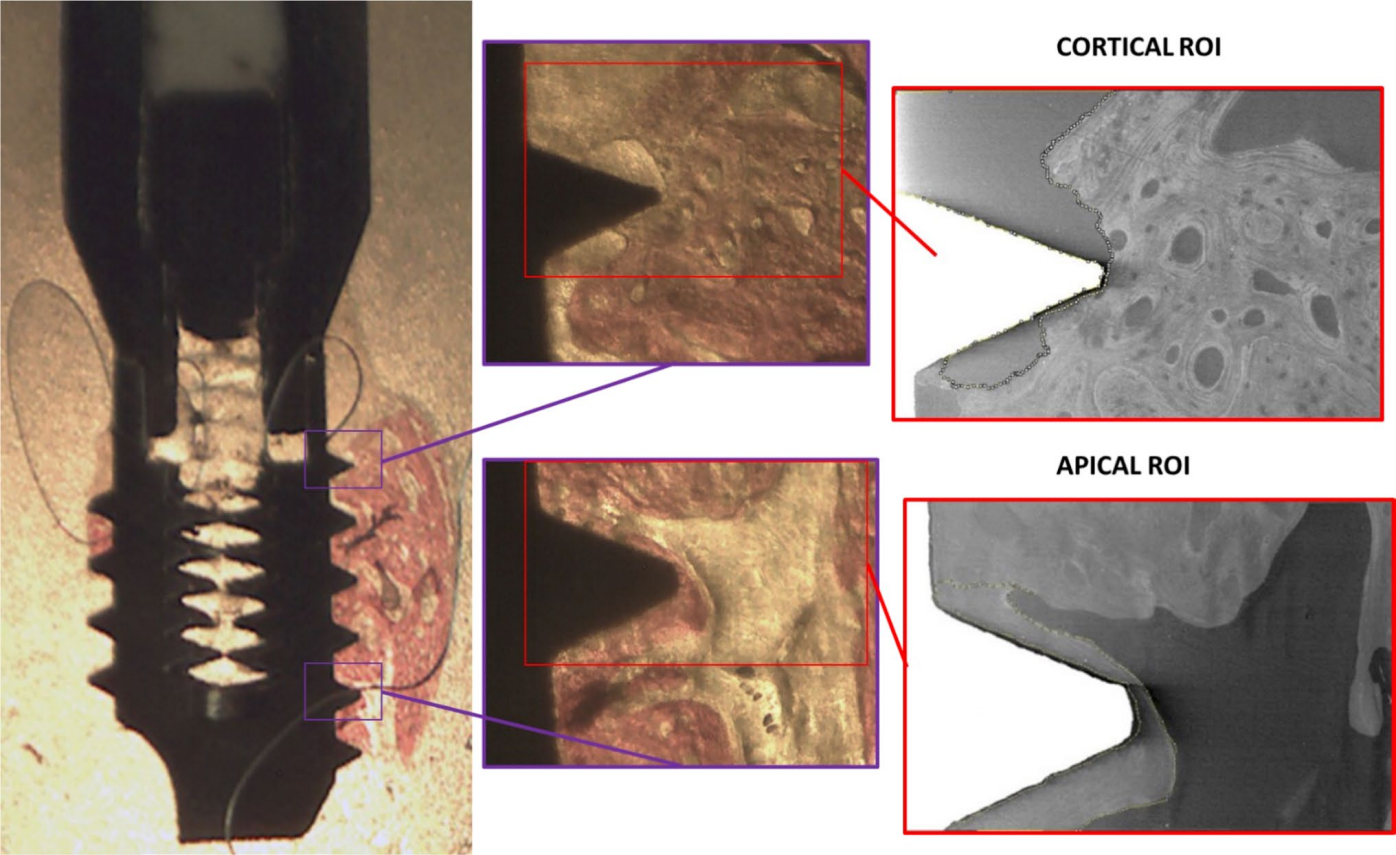
Cortical and apical ROIs selection from a histological sample

Two regions of interest (ROI) of 750 × 500 μm were selected in correspondence to the first cortical (Cortical ROI) and the last apical thread (Apical ROI) with bone tissue (Fig. 1).

**Fig. 2:**
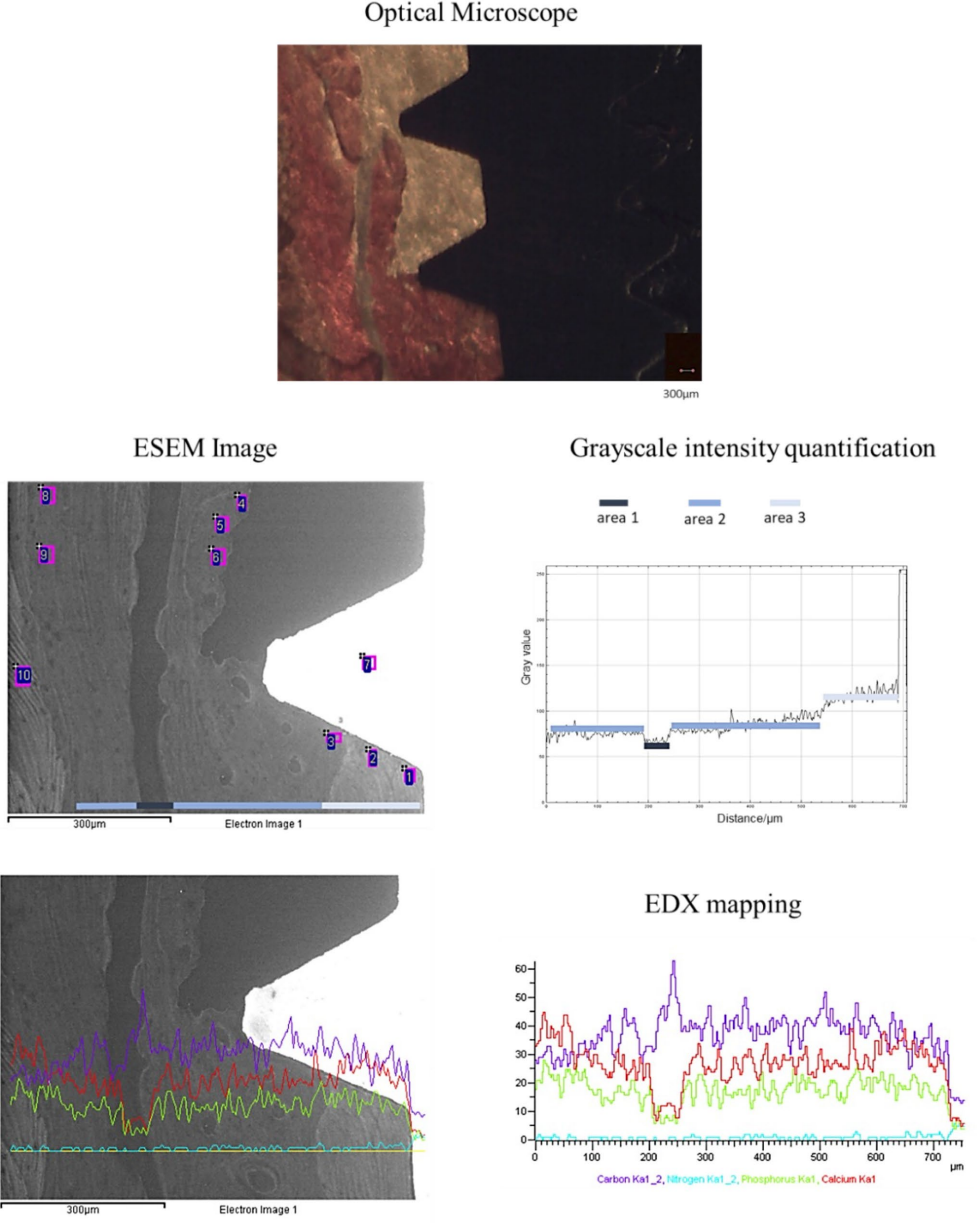
Bone areas calibration performed on the biopsy of one unloaded implant. The area observed at OM (approx. 200× magnification) was analyzed through ESEM and EDX (500× magnification). Greyscale electron density values were then calculated on each image to determine grayscale threshold. Three different gray scale steps were identified on the analyzed area, each of these corresponded to an area with different gray intensity. These data were correlated to the semiquantitative EDX analysis of the mineral (Ca and P) and organic (N) content

The mineral content was measured using EDX with ZAF correction in areas of approximately 30×30 μm, and the qualitative and semiquantitative element contents (weight % and atomic %) were evaluated. The presence of calcium (Ca), phosphorous (P), nitrogen (N), and relative atomic ratios (Ca/P, Ca/N, and P/N) were calculated for all spectra. EDX mapping was also performed to detect the elemental distribution of Ca, P, N, and C in the selected ROIs (Fig. 2).

In each ESEM image, areas with different gray intensities (electron densities) were identified. The value of gray intensity from dark to light was provided by the Image J software (NIH software, Bethesda, MD, USA) and used to set bone areas with different elemental concentrations and morphologies. Bone 1 corresponds to the lowest electron density (dark gray), and bone 4 corresponds to the highest electron density (light gray) (Fig. 2). EDX mapping was also performed to analyze the mineralization gradient (Fig. 2). The grayscale intensity was then correlated with the EDX data following a previous methodology [32] that was applied in other histological biopsy analyses [24, 25].

The extension of different electron-dense bone areas was measured, and the scale bar provided by ESEM was used for ImageJ calibration. Three measurements were performed for each ROI and the mean values (μm2) were recorded. For each ROI, the bone area percentage was calculated.

Analyses of distant bone areas were performed to assess mineralization of the mature cortical bone.

The following bone areas were identified through ESEM/EDX based on grayscale intensity and organic/inorganic ion content (Fig. 2):

- Bone 1: Low-electron-density areas. ESEM images appear as dark gray areas, indicating a low mineralized bone or bone marrow area characterized by low inorganic (Ca, P) and high organic (N) content.
- Bone 2: Medium-electron-density areas. ESEM images appeared as medium-gray areas with numerous lacunae. These areas were defined as remodeled bone, characterized by moderate inorganic (Ca, P) and organic content (N).
- Bone 3: High-electron-density areas. Highly mineralized areas with high Ca, P, and N. Light gray areas are defined as mature old bone and are characterized by high inorganic and low organic contents.
- Bone 4: High-electron-density areas. Highly mineralized areas with a dense and homogeneous structure with high Ca and P. Light gray with high Ca/N and P/N and Ca/P areas were defined as the cortical bone tissue (Table 1).

**Table 1:** EDX atomic values (mean±SD) of Ca, P and N, Ca/N, P/N, Ca/P on the analyzed bone. In the vertical column, significant differences (different letters, *p*<0.05) among bone types are indicated

	Ca	P	N	Ca/N	P/N	Ca/P
Bone 1 Dark gray	1.10±0.5a	0.85±0.4a	13.63±2.3a	0.07±0.05a	0.06±0.04a	1.18±0.03a
Bone 2 Medium gray	1.52±0.6b	1.21±0.5b	12.59±1.6a	0.13±0.06b	0.10±0.05b	1.25±0.03b
Bone 3 Light gray	2.32±0.9c	1.72±0.6c	10.08±1.5b	0.21±0.07c	0.15±0.05c	1.34±0.04c
Bone 4 Light gray	2.78±0.7d	2.50±0.5d	11.68±1.2b	0.24±0.06c	0.19±0.04d	1.45±0.07d

### Statistical analysis

Statistical data were analyzed using SigmaPlot software (Systat Version 13.0, USA).

One-way ANOVA was performed to detect statistically significant differences in the atomic values content and ratios among the bone types. Two-way ANOVA followed by a Holm-Sidak test (normality test *p* > 0.05, equal variance test *p* > 0.05) was performed to detect statistically significant differences between loaded and unloaded biopsies in both coronal and apical ROIs. The *p* value was set at 0.05.

## Results

**Fig. 3:**
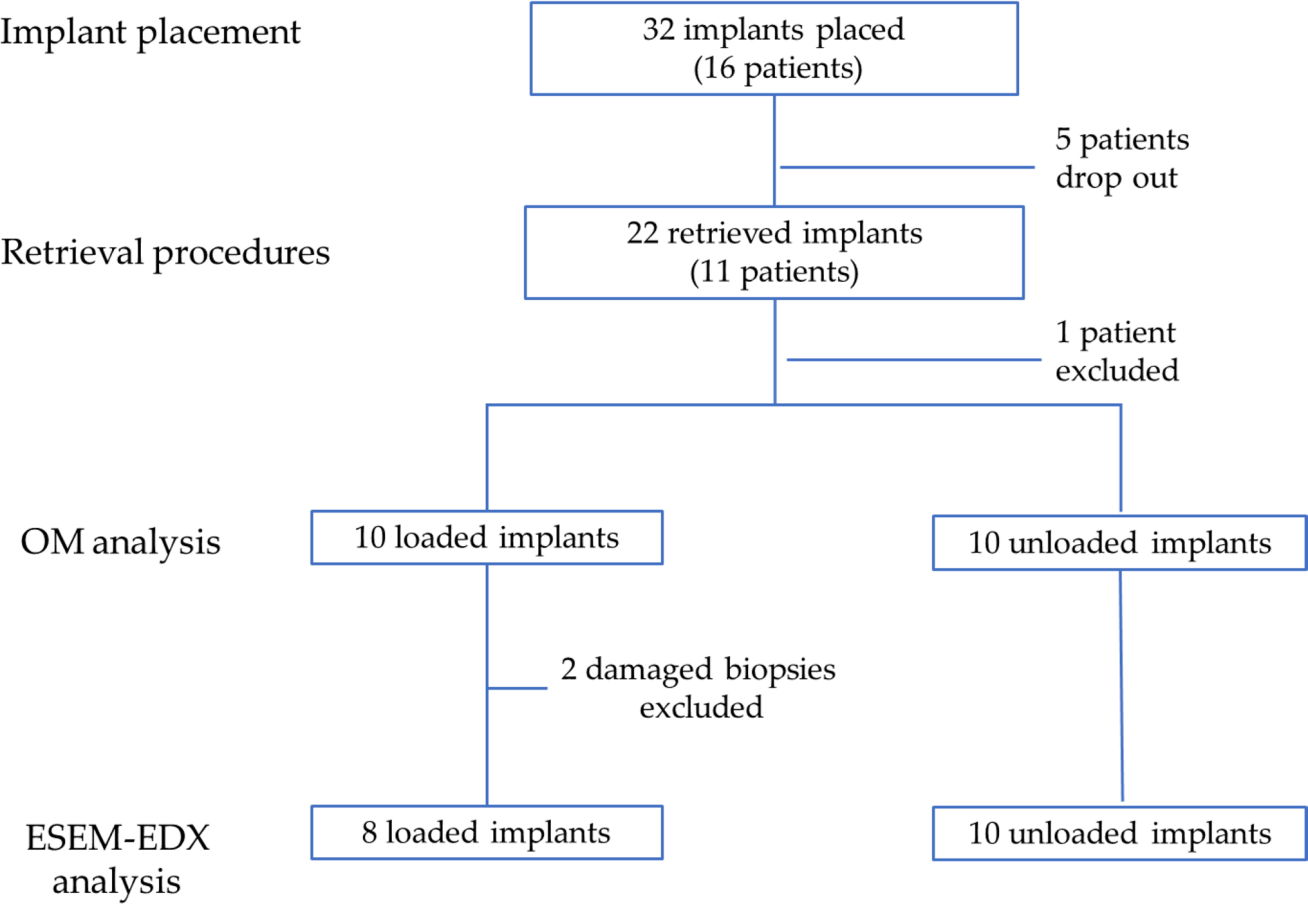
Flowchart of the study. ESEM-EDX was performed on 18 implant biopsies, 8 comprising a loaded implant, 10 comprising an unloaded implant

Five patients were excluded from the study because of chikungunya viral infections. One patient was excluded due to biopsy damage during histological processing. Other two biopsies of loaded implants from two patients were not analyzed because of histological slide detachment. A total of 18 implant biopsies were analyzed: ten included one unloaded implant and eight included one loaded implant (Fig. 3). Two representative histological samples, one from unloaded and one from loaded group are depicted in Figure S1, Supplementary material.

### OM and ESEM-EDX analysis of loaded implants

**Fig. 4:**
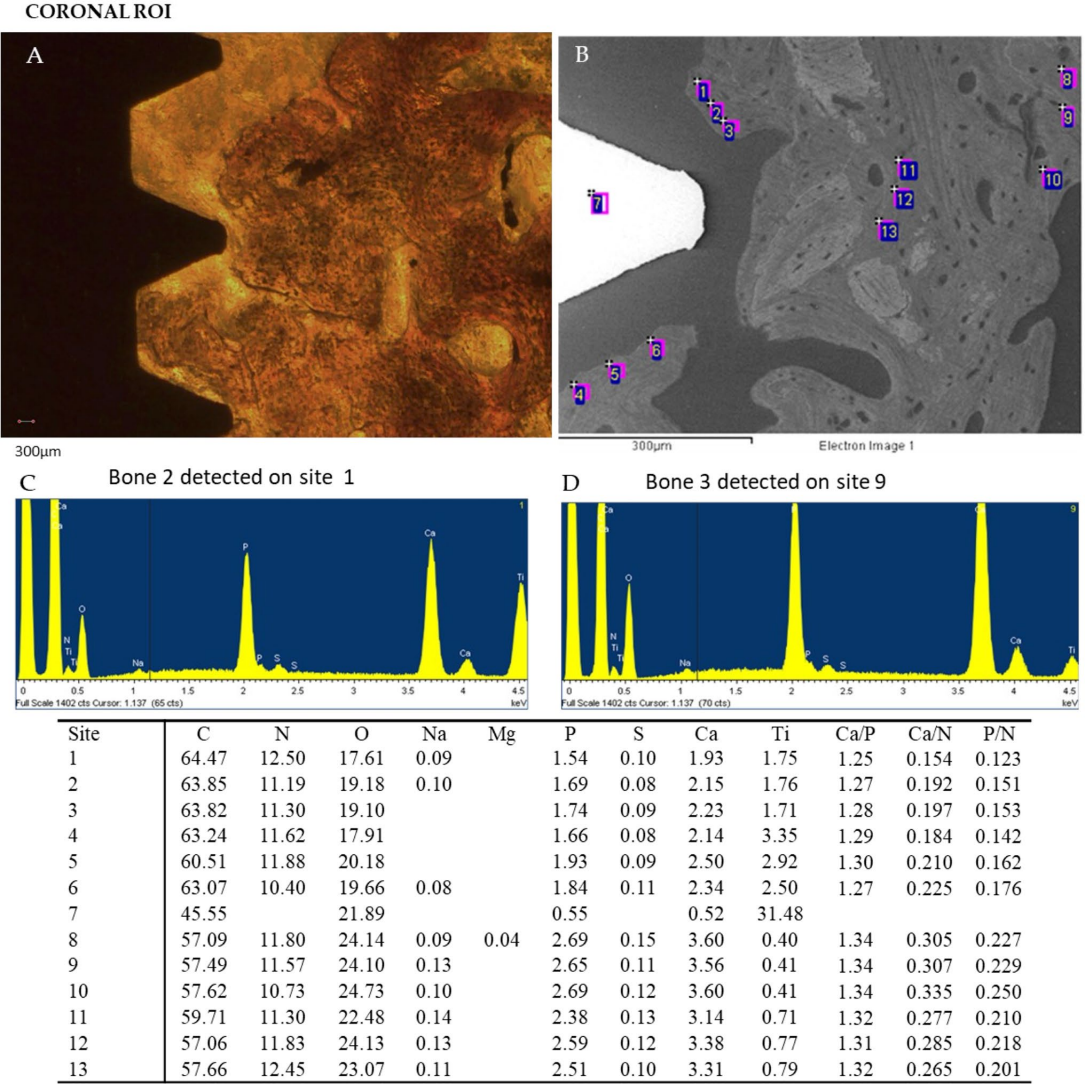
Representative **A** OM and **B** ESEM images of one loaded implant. Scale bar (300 μm) is reported for comparison in both images. **E** EDX analysis performed on the ESEM-image. Values are expressed as atomic percentages. Representative spectra of **C** bone 2 and **D** bone 3 are reported

**Fig. 5:**
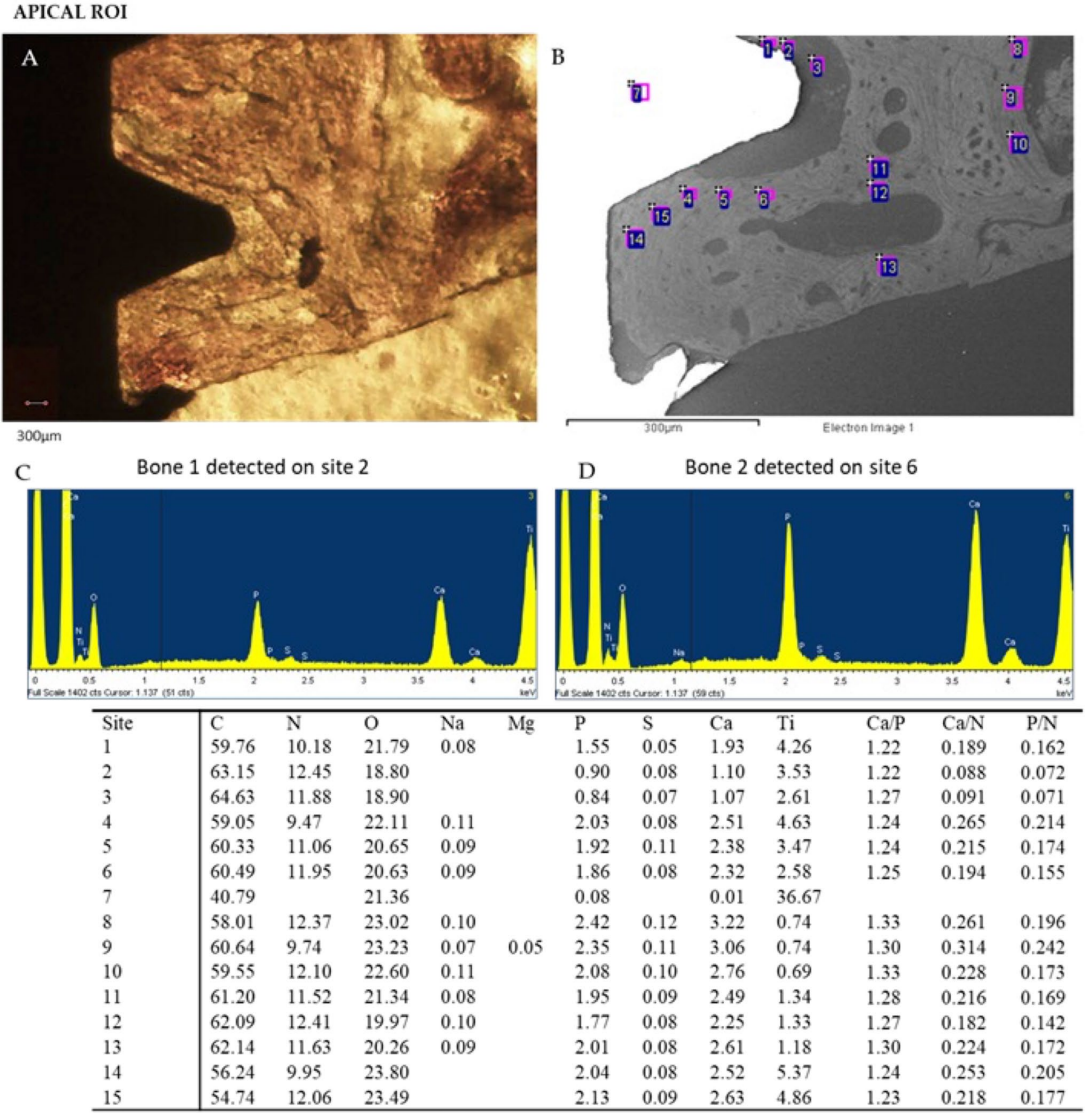
Representative **A** OM and **B** ESEM images of one loaded implant Apical ROI. Scale bar (300 μm) is reported for comparison in both images. **E** EDX analysis performed on the ESEM-image. Values are expressed as atomic percentages. Please note the lower peaks of Ca and P in the spectra of **C** bone 1 when compared to D bone 2

**Fig. 6:**
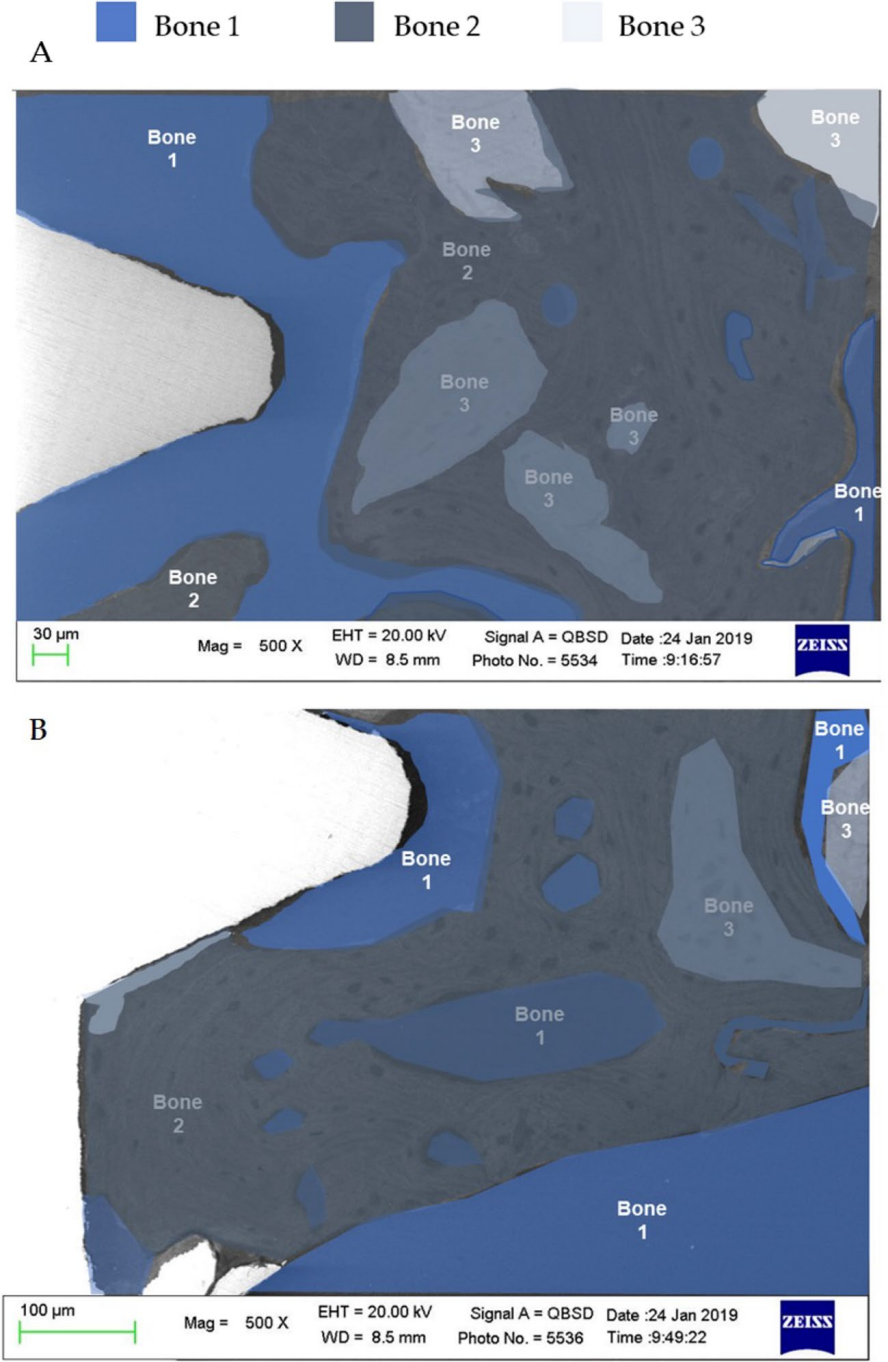
Bone type classification in the selected **A** cortical and **B** apical ROI

Representative OM and ESEM images of cortical and apical ROIs from one loaded implant are reported in Figs. 4 and 5 respectively. Bone areas division/identification is reported in Fig. 6.

At Cortical ROI, low mineralized area was generally observed close to the implant surface (bone 1). Medium mineralized areas were detected approx. 300 μm from the implant surface (bone 2). High electron dense areas (bone 3) were detected close to bone 2 and at the limits of Cortical ROI (approx. 500 to 750 μm from the implant surface).

At the apical ROI, low mineralized areas (bone 1) were uniformly distributed. Medium mineralized area (bone 2) was detected close to the implant surface, while highly mineralized areas were observed at the limits of the ROI (bone 3).

EDX spectra showed the mineral content of the different bone areas in the coronal and apical ROIs (Figs. 4 and 5).

### OM and ESEM-EDX analysis of unloaded implants

**Fig. 7:**
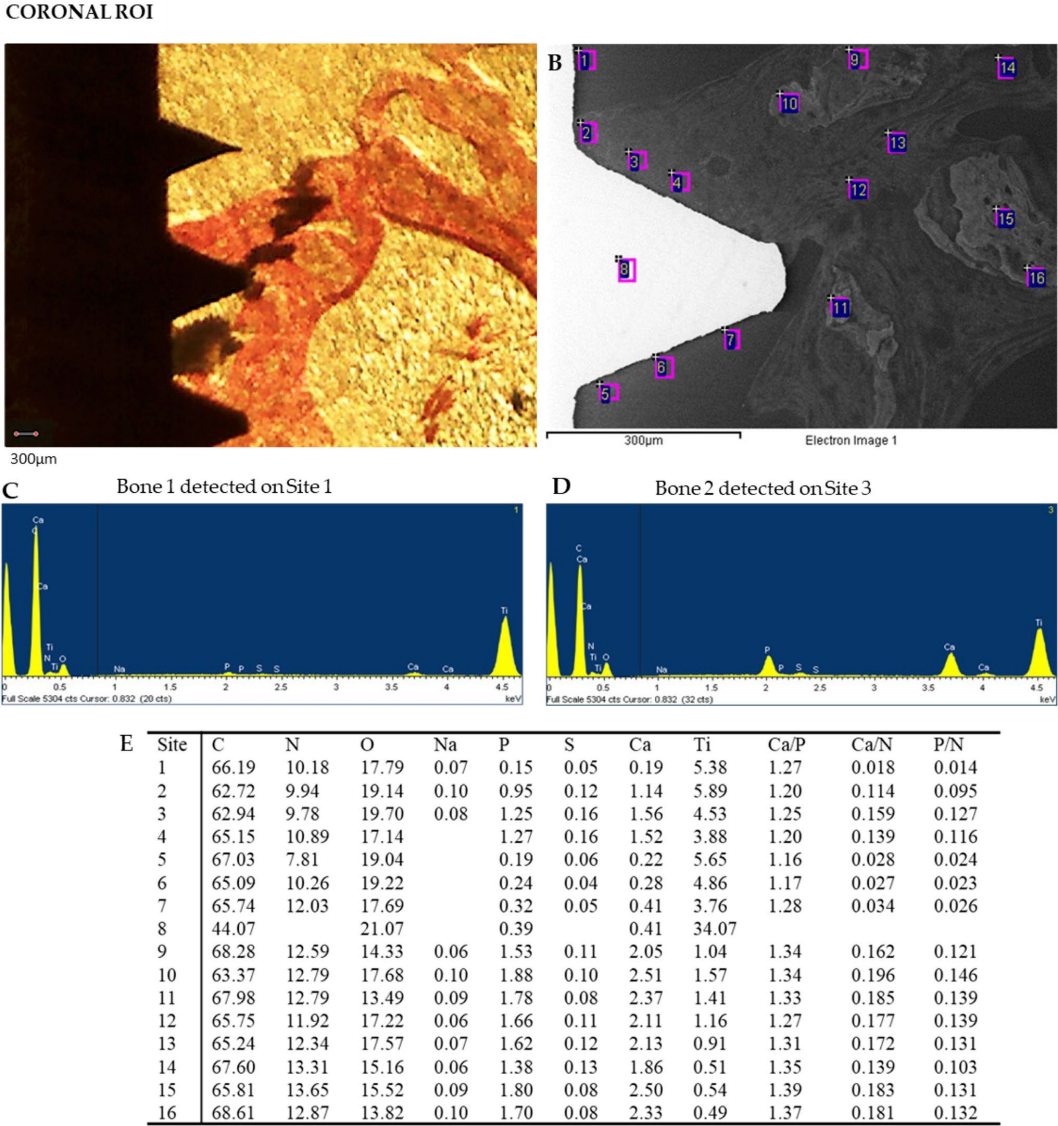
Representative **A** OM and **B** ESEM images of 1 unloaded implant. Scale bar (300 μm) is reported for comparison in both images. **E** EDX analysis performed on the ESEM-image. Values are expressed as atomic percentages. Please note the higher Ca and P peaks in the EDX spectra of **C** bone 3 when compared to (**D**) bone 1

**Fig. 8:**
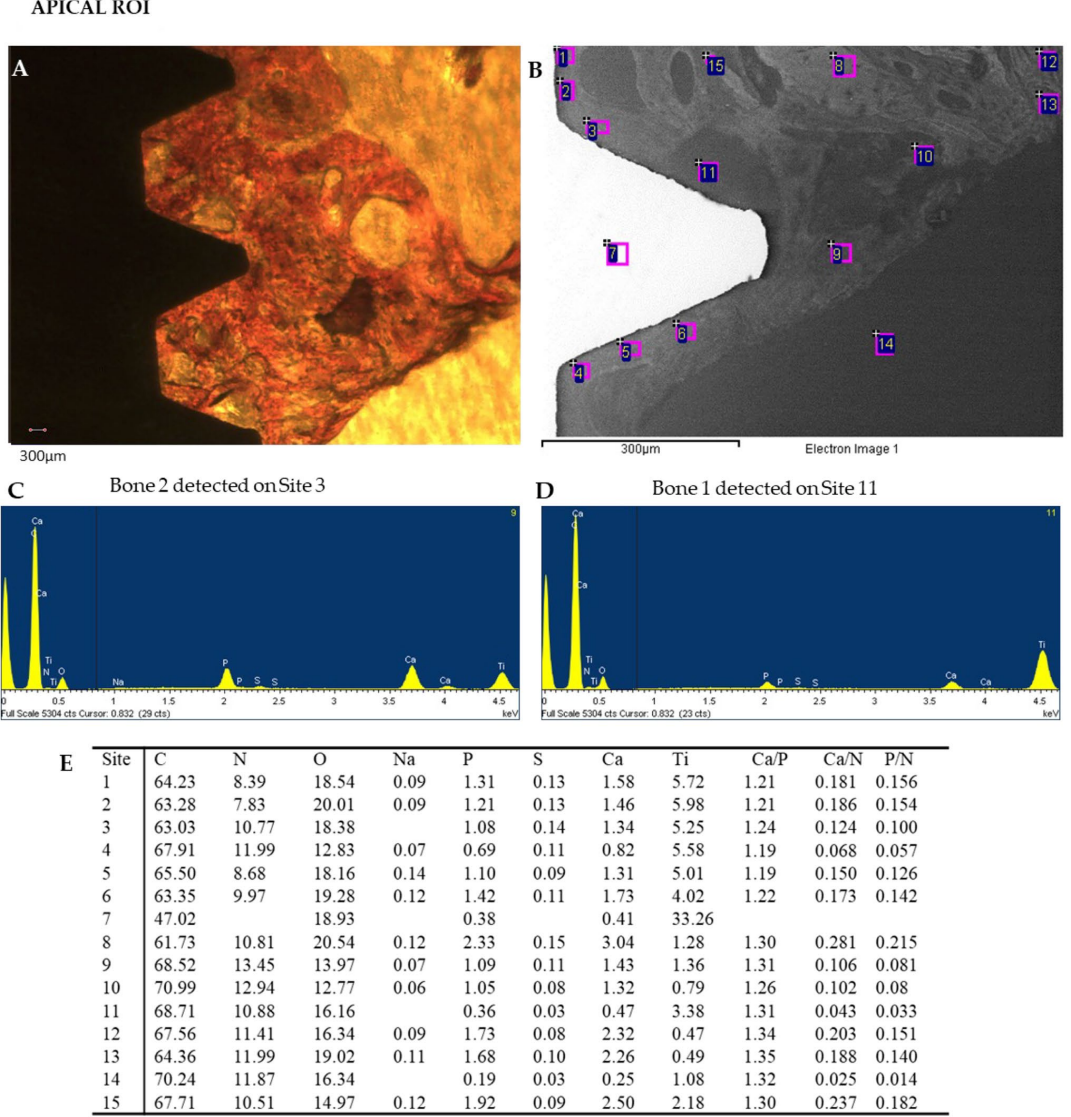
Representative **A** OM and **B** ESEM images of 1 unloaded implant apical ROI. Scale bar (300 μm) is reported for comparison in both images. **E** EDX analysis performed on the ESEM-image. Values are expressed as atomic percentages. Please note the higher EDX peaks of Ca and P of **C** bone 2 when compared to D bone 1

**Fig. 9:**
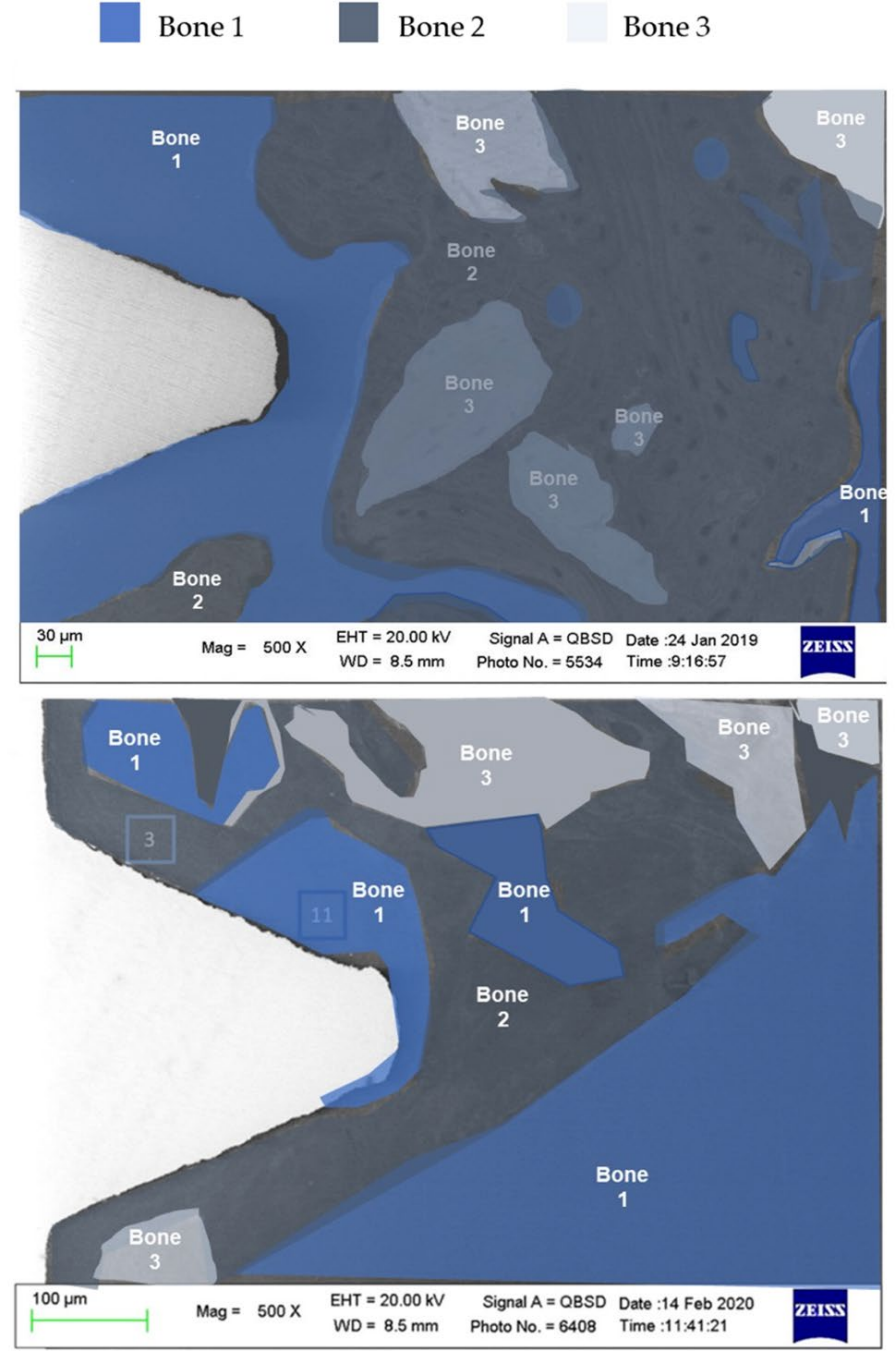
Bone areas division/identification in the selected cortical and apical ROI of the unloaded implant

Representative OM and ESEM images of one loaded implant cortical and apical ROIs are reported in Figs. 7 and 8 respectively. Bone areas division/identification is reported in Fig. 9.

At Cortical ROI, low electron dense areas (bone 1) were mostly detected in sites close to the implant surface, while medium electron dense areas (bone 2) were predominant close to the implant surface and at distant sites. High electron dense areas were mostly observed at sites over 500 μm from the implant surface.

At apical ROI, a higher presence of bone 1 was observed, with limited bones 2 and 3.

### Bone areas distribution in the entire ROIs

The distribution of bone areas in the entire ROIs is reported in Fig. 8 and Table 2.

**Table 2:** Percentages of bone areas (mean± SD) measured at cortical ROI and apical ROI after 4 months from implant placement. In the horizontal row, significant differences (different letters, *p*<0.05) among ROIs are indicated

	Loaded *n*=8		Unloaded *n*=10	
	Cortical ROI	Apical ROI	Cortical ROI	Apical ROI
Bone 1	20.1±7.8a	25.6±15.1a	35.6±14.8b	42.9±18.1b
Bone 2	51.6±12.0a	54.7±8.3a	38.9±11.1b	34.6±12.1b
Bone 3	28.2±11.1a	18.3±12.1a	25.1±10.3a	19.5±9.6a

**Fig. 10:**
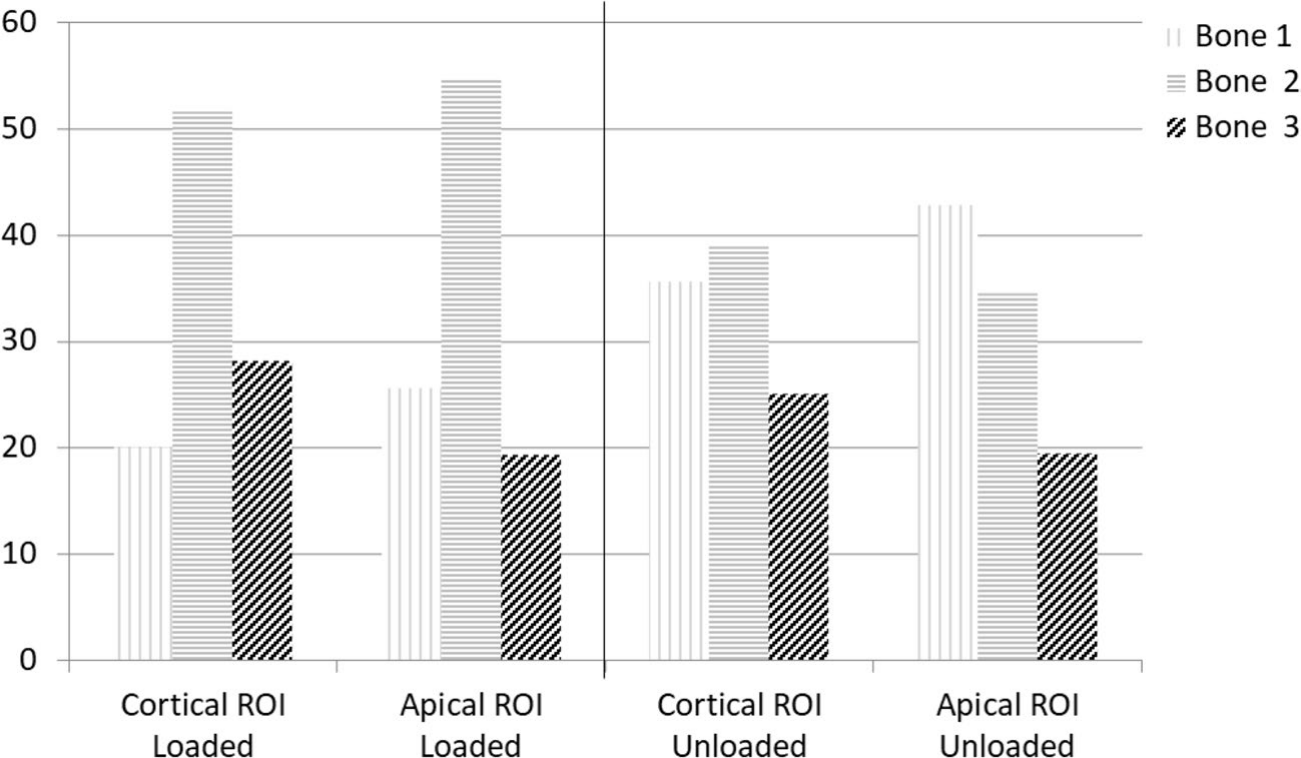
Bone areas distribution in loaded and unloaded implants at the selected ROIs. A high presence of bone 2 was observed in both cortical and apical ROIs. Differently, unloaded implants showed a higher presence of Bone 1

At Cortical ROI, loaded implants showed lower percentages of bone 1 (20.15±7.77) and higher percentages of bone 2 (51.63±12.02) when compared to unloaded implants (percentages of bones 1 and 2 were 35.6±14.8 and 38.9±11.1 respectively) (Table 2). Bone 3 showed similar values in both loaded and unloaded implants. This trend was observed also at the Apical ROI, where significantly lower percentages of bone 1 and higher percentages of bone 2 were detected in loaded implants (Fig. 10).

Bone 4 was not detected in cortical and apical ROI (Table 2). Only few samples of loaded group showed this bone area in distant sites from the cortical ROI (in 2/8 of cases).

### Bone area distribution at peri-implant bone interface

The bone area distribution at peri-implant bone surface is reported in Table 2.

At cortical ROI, loaded implants had a high percentage of bone 1 (50.8±17.4), moderate percentage of bone 2 (30.0±12.65) and low presence of bone 3 (18.3±12.1). This distribution is significantly different in the unloaded implants, where lower percentages of bone 1 (36.8±15.8) (*p*<0.05) were detected at cortical ROI (*p*<0.05).

At apical ROI, loaded implants showed low percentages of bone 1 (22.0±17.5), high percentage of bone 2 (65.0±16.5), and low percentages of bone 3 (13.0±11.8).

Apical surface of unloaded implants showed higher percentage of bone 1 (37.5±15.8), lower presence of bone 2 (50.1±12.0), and similar presence of bone 3 (13.4±8.6). No presence of bone 4 was detected at the interface in any cases.

## Discussion

This study analyzed the bone tissues around loaded or unloaded human-retrieved implants and their level of tissue mineralization. It was focused on a portion of the peri-implant bone of approximately 500–700 μm of thickness, which was defined as the ROI. This region corresponded to the dimensions of the biopsy specimen.

The possibility of obtaining retrieved human biopsies and histological sample preparations allowed a “photography” of the morphological situation and mineral composition of bone 4 months after implant placement. The analysis was performed on histological samples using an ESEM connected to EDX [22, 23]. Mineralization analyses by ESEM-EDX on bone histological biopsies has been innovatively proposed and validated by Gandolfi et al. for bone biomaterials in animal models [22] and for bone around retrieved implants in human [23–25].

The detection of different grayscale intensity areas (by ESEM) was associated with the calculation of organic (low z number elements, such as N) and inorganic (high z number elements, such as Ca and P) atomic percentages and ratios (by EDX) [22–25], allowing the creation of a map just around the implant (in the ROI) to compare with the morphological analyses previously performed by optical microscopy [24, 25].

This study identified different bone areas characterized by different levels of mineralization and different morphologies.

Bone architecture around the implant in the ROI underwent several modifications after the initial loading, in accordance with previous studies [12, 33–38].

In the selected ROIs, a dynamically active newly formed tissue was observed, which envelops and wraps all the implants creating a 400–700-μm thick nest/tissue. This tissue contained areas of moderate/high mineralization (bones 2 and 3, respectively) mixed with areas of low mineralization but rich in protein/collagen (bone 1), as demonstrated by the low Ca/N and P/N ratios. The low-mineralized areas showed high vascularization (detected by optical microscopy). In the periphery of ROIs, a dense mineralized bone was classified as bone 4 and probably represented the sound “old” bone not affected by the biological modification induced by implant site preparation and implant placement.

The presence of different bone areas creates a puzzle that may well represent the active modification that takes part in the first months after placement [8, 35–41]. This bone structure is not far from the concept of honeycomb structure. The region around the implant (in this study, the ROI) contained different tissues with different elasticities and stiffnesses that can be compressed (and released) during mechanical repetitive loading. Accordingly, a recent study reported that the interface between the implant and bone is highly dynamic and “evolves” through a more mineralized and stiff structure [42]. This is probably the biological reason for the different mineralized regions.

The study demonstrated that after 4 months, the presence of bones 3 and 4 at the cortical level was very modest and lower when considering the bone in contact with the implant interface. Bone 4, in particular, was detected only on distant sites from the investigated ROI and in a limited number of specimens. New bone mineralization, formation of bones 2 and 3, started from the apical area and grew from deeper bone to superficial/cortical bone, contributing to the creation and maturation of cortical bone around the implant emergency. These findings suggests that the formation of a sound and complete cortical bone requires additional time.

The effect of implant loading on peri-implant bone was also analyzed in this study. Interestingly, a 2-month loading period induced a marked difference in terms of bone mineralization when compared with unloaded implants. The bone in strict contact with the implant surface showed interesting and unexpected results. In the cortical ROI, the unloaded group showed a larger percentage of bone 2 thighs with implant surface, while the loaded group had a higher percentage of bone 1, the less mineralized bone type.

Implant loading induced the formation of a less mineralized but more elastic structure, represented by thin layers of low-mineralized bone structures that may reduce occlusal trauma and loading stress on healing tissue. During implant loading, the apical ROI underwent higher stress. In this way, the bone remodeling activities started from the most apical ROI and continued to the cortical ROI, which underwent fewer mechanical stimuli at the interface. A previous study reported that under physiological ranges (tolerated micromotion between 50 and 150 μm) [43], mechanical stimulation (i.e., loading) of implants decreases osteoclast activator signaling molecules (i.e., osteoprotegerin), leading to the differentiation of mesenchymal stem cells into osteoblasts and favoring new bone apposition [43]. Bone tissue acts as an elastic nest/chamber that can be deformed by loading, resulting in a reduced mineralized bone in contact with the cortical ROI [44].

The percentage of bone 3 (higher mineralization) in close contact with the implant surface was similar in both the groups. These percentages are probably sufficient to prevent dislocation and excessive movement of the implant.

The limitations of this study were the use of small-diameter implants. The dimensions of the implant were selected to allow easy removal of the implant with a limited residual bone defect [5]. The reduced dimension may be responsible for the observed bone morphology, as many studies have demonstrated that the greater the diameter, the lower the MBL [45, 46]. It should be highlighted that an early loading protocol was performed in the loading group. The loading protocol has been scientifically validated in a previous meta-analysis [31] but could lead to some discrepancies to the clinical practice, where definitive load is usually applied after 6 months.

The present results are not completely comparable with those reported by a previous histomorphometric study, which found only a slightly higher bone implant contact in loaded implants than in unloaded implants [5]. In the present study, only two different regions of interest were evaluated at the cortical and apical levels. Therefore, the percentage of bone in contact with the implant surface varied with previously published data that analyzed the entire bone implant contact.

## Conclusions

The study demonstrated that at least three different bone areas were identified on the basis of different mineralization levels and colors at ESEM in close contact with the implant surface and in the ROI. Four months after placement, the unloaded implants showed a more homogeneous distribution of bone 1, the less mineralized tissue, while the 2-month loaded implant showed more mineralized bone 2 in the ROI.

### Supplementary information

ESM 1 Figure S1. Two representative histological samples, one from unloaded and one from loaded group. In the unloaded implant groups, the cover screws were maintained throughout the 4-month follow-up period. Differently, in the loaded group, abutments and cemented crowns were placed 2 months after insertion and remained *in situ* until the 4-month follow-up. (PNG 4792 kb)

High resolution image (TIFF 35783 kb)

## Data Availability

Data are available on reasonable request.
