# Supplementary figures and images for: Mineralization and morphology of peri-implant bone around loaded and unloaded dental implants retrieved from the human mandible
Source: Oral Maxillofac Surg. 2023 Sep 4;28(2):623–37. doi: 10.1007/s10006-023-01175-1 (PMC11144681; doi:10.1007/s10006-023-01175-1)

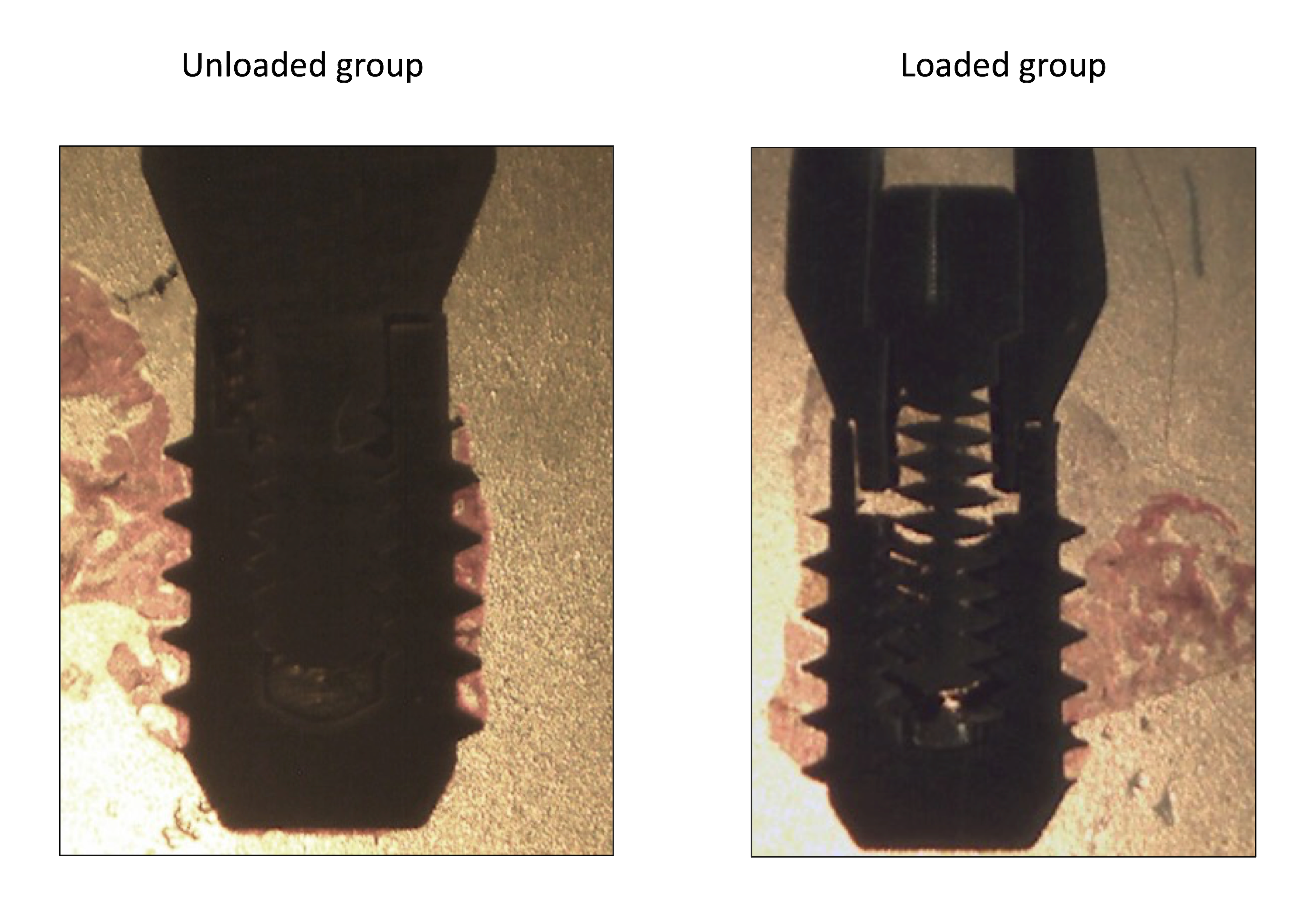

Supplement: Supplementary file 1 — Figure S1. Two representative histological samples, one from unloaded and one from loaded group. In the unloaded implant groups, the cover screws were maintained throughout the 4-month follow-up period. Differently, in the loaded group, abutments and cemented crowns were placed 2 months after insertion and remained in situ until the 4-month follow-up. (PNG 4792 kb) [file 10006_2023_1175_Fig11_ESM.png]

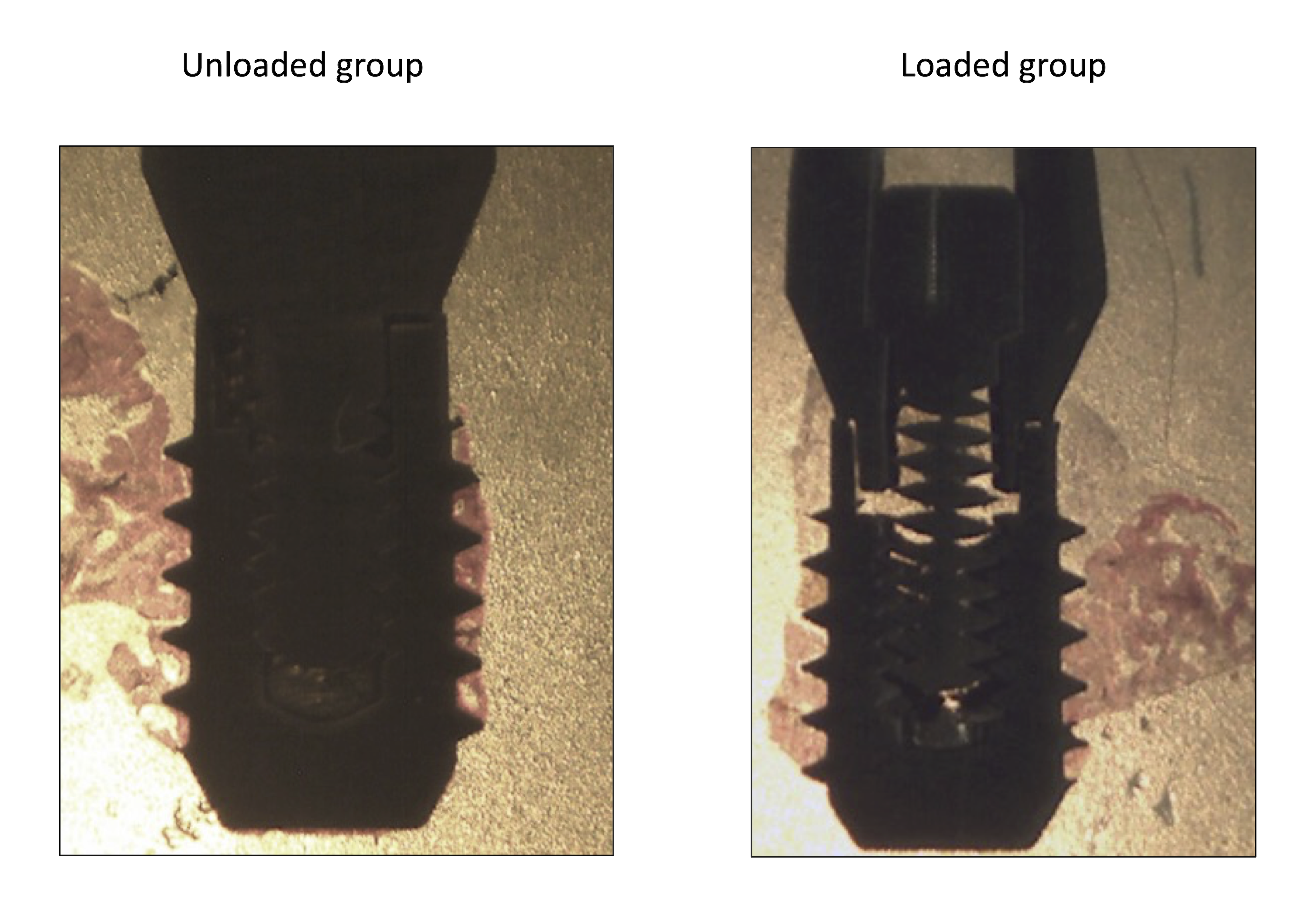

Supplement: Supplementary file 2 — High resolution image (TIFF 35783 kb) [file 10006_2023_1175_MOESM1_ESM.tiff]
